# Assessment of Factors Associated With Perceived Health Anxiety Among Health Profession Medical Students in the Central Region of Saudi Arabia: A Cross‐Sectional Study

**DOI:** 10.1002/hsr2.72981

**Published:** 2026-09-22

**Authors:** Eman Dawood, Agnes Monica, Alyia Abdullah, Ghada Alalyani, Nada Alzhrani, Fai Alqahtani, Ghadeer Bin Ali, Hanaa Abo Shereda

**Affiliations:** ^1^ College of Nursing King Saud Bin Abdulaziz University for Health Sciences Riyadh Saudi Arabia; ^2^ King Abdullah International Medical Research Center Riyadh Saudi Arabia; ^3^ Ministry of National Guard Health Affairs Riyadh Saudi Arabia; ^4^ Department of Psychiatric and Mental Health Nursing, College of Nursing, Shebin‐Elkom Menofyia University Al Minufiyah Egypt

**Keywords:** cross‐sectional study, health anxiety, health profession students, Saudi Arabia, short health anxiety inventory

## Abstract

**Background and Aims:**

Health anxiety may affect and impair well‐being, reassurance‐seeking behavior, healthcare use, and academic functioning among health profession medical students. This study aimed to estimate the prevalence of clinically significant health anxiety and examine sociodemographic, academic, and health‐related factors associated with Short Health Anxiety Inventory (SHAI) scores among health profession students in Riyadh, Saudi Arabia.

**Methods:**

A cross‐sectional study was conducted at King Saud bin Abdulaziz University for Health Sciences, Riyadh, during January–March 2024. Using a convenience sampling technique, an anonymous online questionnaire was completed by 424 students aged 18 years or older, and it included demographic and background characteristics and the 18‐item SHAI, in which clinically significant health anxiety was defined as SHAI score ≥ 27. Descriptive and analytical statistics were applied, with *p* < 0.05 considered statistically significant.

**Results:**

The 424 participants reported a mean age of 20.92 ± 2.07 years, and 351/424 were female (82.8%). The 18‐item SHAI showed good reliability with Cronbach's *α* of 88.3. Clinically significant health anxiety was identified in 77/424 students (18.2%; 95%CI, 14.8%–22.1%). Higher mean SHAI scores were observed among females, students with previous medical or mental health conditions, occasional smokers, students with a first‐degree relative doctor, and students reporting physician visits. In adjusted logistic regression, clinically significant health anxiety was independently associated with choosing college because of a close relative's medical/mental health condition (OR = 2.98; 95%CI, 1.03–8.65; *p* = 0.044), occasional smoking (OR = 4.21; 95% CI, 1.24–14.26; *p* = 0.021), previous medical or mental condition (OR = 2.10; 95%CI, 1.10–4.00; *p* = 0.024), and (OR = 2.61; 95%CI, 1.35–5.04; *p* = 0.004) respectively.

**Conclusion:**

The clinically significant health anxiety was prevalent with nearly one in five health profession students reported clinically significant health anxiety. The findings highlight the need for student‐centered screening, counseling, and longitudinal research.

## 1 Background

Health anxiety is one of the most common types of anxiety, and it refers to excessive worry about having or developing a serious illness and often accompanied by heightened attention to bodily sensations, misinterpretation of benign symptoms, repeated reassurance seeking, checking behavior, or avoidance of health‐related information and settings. Severe and persistent health‐related preoccupation overlaps with illness anxiety disorder and related somatic‐symptom presentations, whereas milder health concerns may represent a normal adaptive response to perceived threat [1, 2, 3]. For assessing health anxiety and hypochondriacal concerns in clinical and nonclinical samples, the Short Health Anxiety Inventory (SHAI) is considered one of the most widely used self‐report measures [2, 3].

There are many consequences to Health anxiety like it can impair quality of life, increase psychological distress, interfere with daily activities, and contribute to unnecessary reassurance seeking or healthcare use. At the public health level, anxiety disorders represent a substantial burden worldwide, and national epidemiological work in Saudi Arabia has reported the prevalence of panic disorder to be 1.6%, that of generalized anxiety disorder was 1.9%, and that of social anxiety disorder was 5.6% highlighted the benefits of mental health surveillance and service planning [4, 5]. The main concern among young adults and university students is that anxiety‐related symptoms may emerge during a developmental period characterized by identity formation, academic transition, and appearance of being responsible.

Due to demanding academic workloads, frequent examinations, high expectations, competitive learning environments, early exposure to illness and suffering, and preparation for future clinical responsibility, health profession students may be particularly vulnerable to anxiety and psychological distress. Several studies have frequently reported high levels of stress, burnout, anxiety, and other psychological distress indicators [6, 7, 8, 9, 10]. In Saudi Arabia, studies conducted during and after the COVID‐19 period also drew attention to mental health concerns among university and health profession students, including nursing and other health‐related disciplines [11, 12].

Health anxiety is particularly relevant to health profession education as students repeatedly and consistently learn about diseases and their related symptoms, complications, diagnostic tests, and adverse outcomes, and this may lead some students to falsely interpret normal bodily sensations as signs of illness, and this phenomenon has been described in literature as medical student disease, nosophobia, illness anxiety, or hypochondriacal concern [13, 14, 15].

Health anxiety may be associated with several student‐related characteristics, including sex, educational stage whether clinical or preclinical, smoking status, present or past personal and family medical or psychiatric history, familial healthcare professions, and recent healthcare‐seeking behavior, in addition to repeated online symptom checking, which may also reinforce health concerns among students [16]. Studies from several countries like f Peru, the United Arab Emirates, Saudi Arabia, Pakistan, and other settings have investigated and reported varied health anxiety scores shaped by local educational environments, healthcare exposure, cultural expectations, and access to support services among medical students [13, 14, 15, 16, 17, 18].

In Saudi Arabia, despite increasing attention to student mental health, there is scarce evidence on health anxiety among health profession students, specially outside single‐discipline samples. This study aimed to estimate the prevalence of clinically significant health anxiety and examine sociodemographic, academic, and health‐related factors associated with SHAI scores among health profession students in Riyadh, Saudi Arabia, which may encourage and help universities design screening, counseling, and student‐support strategies.

## 2 Materials and Methods

### 2.1 Study Design and Setting

An analytical cross‐sectional study was carried out during January‐March 2024 at King Saud bin Abdulaziz University for Health Sciences (KSAU‐HS), Riyadh, Saudi Arabia after Institutional Review Board approval.

### 2.2 Study Population and Sampling

The students aged 18 years or older enrolled in health profession colleges at KSAU‐HS Riyadh and who voluntarily agreed to participate were included in the study. Using a convenience sampling technique and through the university message center, students from the colleges of Science and Health Professions, Medicine, Nursing, Dentistry, Pharmacy, Public Health, and Applied Medical Sciences were recruited.

### 2.3 Sample Size Calculation

Using Raosoft sample size calculator, the required sample size was calculated based on a finite student population of 4709 at KSAU‐HS Riyadh, and with inputs including a 95% confidence level, an expected response distribution of 50% to provide the most conservative estimate, and a 5% margin of error, the minimum required sample size of approximately 354 participants was yielded but after distribution of the questionnaire, a total of 424 students completed it and were included in the final analysis.

### 2.4 Data Collection Tool

The participants were subjected to an anonymous, self‐administered online English questionnaire including;

1. Sociodemographic, academic, and health‐related background variables, including age, sex, marital status, college, academic year, reason for choosing the current college, smoking status, presence of family history of health profession like a first‐degree relative doctor or healthcare worker, past history of medical condition or mental health condition, and seeking medical advice during the previous 6 months,
2. The 18‐item SHAI, which assesses health anxiety with its two sections assessing illness likelihood section (14 items) and negative consequences Section (Conclusion, 4 items). Total scores range from 0 to 54, a cutoff score of ≥ 27 was used to indicate clinically significant health anxiety. The SHAI has demonstrated strong internal consistency, with Cronbach s alpha ranging from 0.87 to 0.95 [2, 3]. Current‐sample internal consistency was assessed with Cronbach's *α* of 88.3.

### 2.5 Ethical Considerations

Approval was obtained from the CON‐R Research Unit and the Institutional Review Board Committee at King Abdullah International Medical Research Center (KAIMRC; approval number SP23R/242/11). Invitation emails were distributed to all KSAU‐HS Riyadh students via the university message center, including the study purpose and procedures. Informed consent was secured, and students completed the survey anonymously and had the right to withdraw without penalty.

### 2.6 Statistical Analysis

Data was analyzed using IBM SPSS Statistics version 27, where descriptive statistics were used to summarize participants' sociodemographic, academic, and health‐related characteristics. qualitative variables were presented as frequencies, percentages, and 95% confidence intervals, while quantitative variables were presented as mean ± standard deviation (SD). The prevalence of clinically significant health anxiety was calculated using the SHAI cutoff score of ≥ 27. For comparisons of SHAI total score, SHAI main section score, and SHAI negative consequences score across participant characteristics, nonparametric, including Mann–Whitney *U*‐test for two‐group variables, and the Kruskal–Wallis *H*‐test for more than two groups. For Mann–Whitney analyses, the *U*‐statistic, standardized *Z*‐value, *p*‐value, and effect size *r* were reported. For Kruskal–Wallis analyses, the *H*‐statistic, *p*‐value, and epsilon‐squared effect size (*ε* ^2^) were reported. To examine the associations between participant characteristics and clinically significant health anxiety (SHAI ≥ 27), the chi‐square test or Fisher's exact test, were applied as appropriate, with reporting phi (*φ*) or Cramer's *V* as effect‐size measures. Following that, Binary logistic regression was performed with clinically significant health anxiety (SHAI ≥ 27: yes/no) as the dependent outcome. Variables that showed statistically significant bivariate associations with the binary outcome were directly entered into the adjusted model; these comprised reason for choosing college, smoking status, previous medical condition, and previous mental health condition. Adjusted odds ratios (aORs) were used to express the regression results with corresponding 95% CIs and *p*‐values, using Personal interest, nonsmoking, and the absence of previous medical or mental health conditions as the reference categories. All tests were two‐sided, and *p* < 0.05 was considered statistically significant.

## 3 Results

A total of 424 health profession students participated in the study and reported a mean age of 20.92 ± 2.07 years (95%CI, 20.72–21.12). Female represented the major proportion (82.8%; 95%CI, 78.9%–86.1%) and single (96.7%). Most participants were from the College of Nursing (44.6%), followed by Applied Medical Sciences (15.8%), the College of Science and Health Professions (13.9%), and the College of Medicine (13.4%). The selection of the college was mostly based on personal interest (67.0%). The majority of students were nonsmokers (85.1%) with no previous medical condition before starting college (82.5%), or previous mental health condition (84.4%). A total of 55.7% had visited a physician in the last 6 months for various reasons (regular checks, flu, dental problems, allergies, asthma, acne, gastrointestinal tract (GIT) problems, Urinary Tract Infections, eyesight examinations, polycystic ovaries, receiving vaccinations, anxiety, depression, and psychotherapy) versus 55.7% reported no physician visit (Table 1).

**Table 1:** Sociodemographic characteristics of the study sample (*n* = 424).

Variable	*N*	%	95%CI
Gender			
− Female	351	82.8	78.99–86.1
− Male	73	17.2	13.9–21.1
Age: Mean ± SD	20.92 ± 2.07		20.72–21.12
Marital status			
− Single	410	96.7	94.5–98.0
− Married	10	2.4	1.3–4.3
− Divorced	4	0.9	0.4–2.4
College			
− College of Science and Health Professions (COSHP)	59	13.9	10.9–17.5
− Medicine	57	13.4	10.5–17.0
− Nursing	189	44.6	39.9–49.3
− Dentistry	26	6.1	4.2–8.8
− Pharmacy	16	3.8	2.3–6.0
− Public Health	10	2.4	1.3–4.3
− Applied Medical Sciences	67	15.8	12.6–19.6
Academic level			
− Year 1	68	16.0	12.9–19.8
− Year 2	49	11.6	8.9–15.0
− Year 3	90	21.3	17.6–25.4
− Year 4	160	37.7	33.3–42.4
− Year 5	34	8.0	5.8–11.0
− Year 6	23	5.4	3.6–8.0
Reason for choosing your college			
− GPA	102	24.0	20.2–28.3
− Personal interest	284	67.0	62.4–71.3
− Personal medical or mental health condition	16	3.8	2.3–6.0
− Close relative medical or mental health condition	22	5.2	3.5–7.7
Smoking condition			
− Regular smoker	37	8.7	6.4–11.8
− Occasional smoker	13	3.1	1.8–5.2
− Passive smoker	13	3.1	1.8–5.2
− Nonsmoker	361	85.1	81.4–88.2
First‐degree relative doctor?			
− Yes	129	30.4	26.2–35.0
− No	295	69.6	65.0–73.8
First‐degree relative healthcare worker?			
− Yes	187	44.1	39.5–48.9
− No	237	55.9	51.1–60.5
History of medical condition before starting college?			
− Yes	74	17.5	14.1–21.4
− No	350	82.5	87.6–85.9
History of mental condition before starting college?			
− Yes	66	15.6	12.4–19.3
− No	358	84.4	80.7–87.6
Physician visit in the last 6 months?			
− Yes	188	44.3	39.7–49.1
− No	236	55.7	50.9–60.3

Table 2 demonstrate that various variables have a significant correlation, including Sex: females 18.1 ± 8.8 versus males 14.9 ± 8.8 (*U* = 10206.00, *Z* = −2.74, *p* = 0.006, *r* = 0.13), College (*H* = 13.48, *p* = 0.036, *ε* ^2 ^= 0.02) and SHAI consequences score (*H* = 14.66, *p* = 0.023, *ε* ^2 ^= 0.02), with the highest total mean in Pharmacy (22.8 ± 10.9). Smoking status: total SHAI score (*H* = 9.12, *p* = 0.028, *ε* ^2 ^= 0.01) and SHAI main score (*H* = 11.51, *p* = 0.00). Having a first‐degree relative doctor was associated with a higher total SHAI score: 19.2 ± 9.3 versus 16.9 ± 8.7 (*U* = 16206.00, *Z* = −2.43, *p* = 0.015, *r* = 0.12). Previous medical and mental health condition showed the largest effects: 21.7 ± 10.0 versus 16.7 ± 8.4 (*U* = 9230.50, *Z* = −3.89, *p* < 0.001, *r* = 0.19) and 22.9 ± 10.2 versus 16.6 ± 8.3 (*U* = 7556.50, *Z* = −4.66, *p* < 0.001, *r* = 0.23). A physician visit in the last 6 months resulted in a total SHAI score of 19.0 ± 8.7 versus 16.5 ± 8.9 (*U* = 18718.50, *Z* = −2.77, *p* = 0.006, *r* = 0.13). However, the SHAI consequences difference was not significant (*p* = 0.056). The reason for choosing college was associated with a total SHAI score (*H* = 8.41, *p* = 0.038, *ε* ^2 ^= 0.01) and SHAI main score (*H* = 11.76, *p* = 0.008, *ε* ^2 ^= 0.02), which was highest in the close‐relative medical/mental‐health group (23.2 ± 9. Other variables with no significant association included marital status (*H* = 1.52, *p* = 0.467), SHAI main score (*H* = 1.74, *p* = 0.419), or SHAI consequences score (*H* = 0.18, *p* = 0.913), academic level: total SHAI score (*H* = 3.23, *p* = 0.665), SHAI main score (*H* = 5.36, *p* = 0.374), or SHAI consequences score (*H* = 6.67, *p* = 0.246), and having a first‐degree healthcare worker (*p* = 0.194) (Table 2).

**Table 2:** Factors associated with the total short health anxiety inventory (SHAI) score, SHAI main section score, and SHAI negative consequences score (*n* = 424).

Variables	Total SHAI mean score				SHAI main section				SHAI negative consequences			
	Mean ± SD	Test of sig	*p*‐ value	ES	Mean ± SD	Test of sig	*p*‐ value	ES	Mean ± SD	Test of sig	*p*‐ value	ES
Sex												
− Female (351)	18.1 ± 8.8	2.73	0.006*	0.13	14.2 ± 7.3	2.41	0.016*	0.12	3.9 ± 2.7	2.39	0.017*	0.12
− Male (73)	14.9 ± 8.8				11.9 ± 7.4				3.0 ± 2.1			
Marital status												
− Single (410)	17.5 ± 8.7	1.52	0.467	0.00	13.8 ± 7.3	1.74	0.419	0.00	3.8 ± 2.6	0.18	0.913	0.00
− Married (10)	17.4 ± 13.9				13.6 ± 10.7				3.8 ± 3.2			
− Divorced (4)	21.8 ± 12.4				18.5 ± 10.8				3.2 ± 2.2			
College												
− COSHIP (59)	16.6 ± 9.8	13.48	0.036*	0.02	12.5 ± 8.2	10.78	0.096	0.01	4.0 ± 2.5	14.66	0.023	0.02
− Medicine (57)	15.3 ± 8.2				12.5 ± 7.2				2.8 ± 2.2			
− Nursing (189)	17.9 ± 9.2				14.0 ± 7.4				3.9 ± 2.9			
− Dentistry (26)	17.9 ± 9.3				14.9 ± 8.0				3.0 ± 2.1			
− Pharmacy (16)	22.8 ± 10.9				17.8 ± 8.7				5.0 ± 3.3			
− Public Health (10)	19.9 ± 8.8				15.4 ± 6.6				4.5 ± 2.8			
− Applied Medical Sciences (67)	17.9 ± 6.7				14.1 ± 6.0				3.8 ± 1.8			
Academic level												
− Year 1 (68)	17.2 ± 7.8	3.23	0.665	0.00	13.5 ± 6.6	5.36	0.374	0.00	3.7 ± 2.5	6.67	0.246	0.00
− Year 2 (49)	18.3 ± 9.1				13.9 ± 7.5				4.4 ± 2.6			
− Year 3 (90)	18.2 ± 9.1				14.5 ± 7.8				3.7 ± 2.3			
− Year 4 (160)	16.8 ± 8.4				13.1 ± 6.9				3.7 ± 2.8			
− Year 5 (34)	20.2 ± 11.5				16.6 ± 9.3				3.6 ± 2.6			
− Year 6 (23)	16.7 ± 9.9				13.5 ± 7.8				3.2 ± 3.2			
Reason for choosing the college												
− GPA (102)	17.2 ± 8.9	0.038*	0.038*	0.01	13.3 ± 7.6	11.76	0.008*	0.02	3.9 ± 2.4	3.09	0.378	0.00
− Personal interest (284)	17.3 ± 8.7				13.6 ± 7.2				3.7 ± 2.7			
− Personal medical or mental health condition (16)	17.1 ± 9.8				14.3 ± 8.0				2.8 ± 2.3			
− Close relative medical or mental health condition (22)	23.2 ± 9.2				19.0 ± 7.1				4.2 ± 2.7			
Smoking condition												
− Regular smoker (37)	19.6 ± 10.3	9.12	0.028*	0.01	15.5 ± 8.2	11.51	0.009*	0.02	4.1 ± 3.0	1.02	0.797	0.00
− Occasional smoker (13	23.5 ± 0.6				19.4 ± 8.4				4.2 ± 2.6			
− Passive smoker (13)	17.8 ± 8.1				14.4 ± 6.7				3.5 ± 3.0			
− Nonsmoker (361)	17.2 ± 8.7				13.4 ± 7.2				3.7 ± 26			
First‐degree relative doctor?												
− Yes (129)	19.2 ± 9.3	2.43	0.015*	0.12	15.0 ± 7.7	2.27	0.023*	0.11	4.2 ± 2.7	2.20	0.028	0.11
− No (295)	16.9 ± 8.7				13.3 ± 7.2				3.6 ± 2.6			
First‐degree relative healthcare worker?												
− Yes (187)	18.3 ± 9.2	1.29	0.194	0.06	14.5 ± 7.7	1.47	0.141	0.07	3.8 ± 2.7	0.24	0.813	0.01
− No (237)	17.0 ± 8.7				13.3 ± 7.1				3.7 ± 2.6			
History of medical condition before starting college?												
− Yes (74)	21.7 ± 10.0	3.88	< 0.001*	0.19	17.4 ± 8.2	4.08	< 0.001*	0.20	4.3 ± 2.8	1.95	0.052	0.09
− No (350)	16.7 ± 8.4				13.1 ± 7.0				3.6 ± 2.6			
History of mental health condition before starting college?												
− Yes (66)	22.9 ± 10.2	4.65	< 0.001*	0.23	18.0 ± 8.4	4.46	< 0.001*	0.22	4.9 ± 2.6	2.95	< 0.001*	0.19
− No (358)	16.6 ± 8.3				13.1 ± 6.9				3.5 ± 2.6			
Physician visit in the last 6 months?												
− Yes (188)	19.0 ± 8.7	2.76	0.006*	0.13	15.0 ± 7.4	2.79	0.005*	0.14	4.0 ± 2.5	1.90	0.056	0.09
− No (236)	16.5 ± 8.9				12.9 ± 7.3				3.6 ± 2.7			

*Note:* Values are presented as mean ± SD for more description while comparison between‐group comparisons were performed using the Mann–Whitney *U*‐test and the Kruskal–Wallis test for ordinal variables with two or more than two groups respectively For Mann–Whitney *U*‐test, the standardized *Z*‐statistic was reported, and the effect size was expressed as *r* and For the effect size was expressed as epsilon squared (*ε* ^2^). The bold values and * means significant *p* values.

Table 3 demonstrates that clinically significant health anxiety was identified in 18.2% of the students. The proportion was significantly higher among students who selected their college because of a close relative medical/mental health condition (50.0%) compared with those choosing by personal interest (15.1%), GPA (18.6%), or personal medical/mental health condition (25.0%;, *p* < 0.001, Cramer's *V* = 0.20) and also it was higher among occasional smokers (53.8%) compared with nonsmokers (16.3%; *p* = 0.005, Cramer's *V* = 0.17). Students with previous medical conditions had a higher prevalence than those without such history (36.5% vs. 14.3%; *p* < 0.001, Phi = 0.22), and the same way was observed for previous mental health conditions (40.9% vs. 14.0%, *p* < 0.001, Phi = 0.25). Moreover, no statistically significant associations were observed for sex, marital status, college, academic level, first‐degree relative doctor, first‐degree relative healthcare worker, or physician visit in the last 6 months (*p* > 0.05) (Table 3).

**Table 3:** Association between students’ characteristics and clinically significant health anxiety (SHAI ≥ 27).

Variable	SHAI < 27: (No. = 347) *n* (%)	SHAI ≥ 27: (No. = 77) *n* (%)	*χ*2	*p*‐ value	Effect size
Sex					
− Female	64 (87.7)	9 (12.3)	2.02	0.155	Phi = 0.07
− Male	283 (80.6)	68 (19.4)			
Marital status					
− Single	336 (82.0)	74 (18.0)	3.18	0.204	Cramer's *V* = 0.09
− Married	9 (90.0)	1 (10.0)			
− Divorced	2 (50.0)	2 (50.0)			
College					
− COSHP	51 (86.4)	8 (13.6)	9.70	0.138	Cramer's *V* = 0.15
− Medicine	51 (89.5)	6 (10.5)			
− Nursing	149 (78.8)	40 (21.2)			
− Dentistry	20 (76.9)	6 (23.1)			
− Pharmacy	10 (62.5)	6 (37.5)			
− Public Health	8 (80.0)	2 (20.0)			
− Applied Medical Sciences	58 (86.6)	9 (13.4)			
Academic level					
− Year 1	60 (88.2)	8 (11.8)	9.68	0.085	Cramer's *V* = 0.15
− Year 2	40 (81.6)	9 (18.4)			
− Year 3	72 (80.0)	18 (20.0)			
− Year 4	135 (84.4)	25 (15.6)			
− Year 5	22 (64.7)	12 (35.3)			
− Year 6	18 (78.3)	5 (21.7)			
Reason for choosing your college					
− GPA	83 (81.4)	19 (18.6)	17.27	< 0.001*	Cramer's *V* = 0.20
− Personal interest	241 (84.9)	43 (15.1)			
− Personal medical or mental health condition	12 (75.0)	4 (25.0)			
− Close relative medical or mental health condition	11 (50.0)	11 (50.0)			
Smoking condition					
− Regular smoker	28 (75.7)	9 (24.3)	12.95	0.005*	Cramer's *V* = 0.17
− Occasional smoker	6 (46.2)	7 (53.8)			
− Passive smoker	11 (84.6)	2 (15.4)			
− Nonsmoker	302 (83.7)	59 (16.3)			
First‐degree relative doctor?					
− Yes	248 (84.1)	47 (15.9)	3.24	0.072	Phi = 0.09
− No	99 (76.7)	30 (23.3)			
First‐degree relative healthcare worker?					
− Yes	200 (84.4)	37 (15.6)	2.35	0.125	Phi = 0.07
− No	147 (78.6)	40 (21.4)			
History of medical condition before starting college?					
− Yes	300 (85.7)	50 (14.3)	20.26	< 0.001*	Phi = 0.22
− No	47 (63.5)	27 (36.5)			
History of mental condition before starting college?					
− Yes	308 (86.0)	50 (14.0)	27.22	< 0.001*	Phi = 0.25
− No	39 (59.1)	27 (40.9)			
Physician visit in the last 6 months?					
− Yes	200 (84.7)	36 (15.3)	3.02	0.082	Phi = 0.08
− No	147 (78.2)	41 (21.8)			

*Note:* * Means significant *p* value.

Table 4 displays the adjusted binary logistic regression model, which selected the factors that were statistically associated with clinically significant health anxiety, and the findings show that when compared to choosing a college based on a close relative medical/mental health condition (OR = 2.98, 95% CI, 1.03–8.65, *p* = 0.044). Occasional smoking was also associated with higher odds compared to nonsmokers (OR = 4.21, 95% CI, 1.24–14.26, *p* = 0.021), whereas regular smoking and passive smoking were not statistically significant. Students with a previous medical condition had approximately twice the odds of clinically significant health anxiety (OR = 2.10, 95% CI, 1.10–4.00, *p* = 0.024), and those with a previous mental health condition had the highest odds as well (OR = 2.61, 95% CI, 1.35–5.04, *p* = 0.004).

**Table 4:** Binary logistic regression for factors associated with high SHAI ≥ 27.

Predictor	Comparison category	adjusted OR	95% CI	*p*‐ value
Reason for choosing college	Personal interest (Ref)			
	GPA	0.77	0.41–1.46	0.428
	Personal medical/mental health condition	1.11	0.29–4.27	0.881
	Close relative medical/mental health condition	2.98	1.03–8.65	0.044*
Smoking condition	Nonsmoker			
	Regular smoker	1.31	0.55–3.12	0.537
	Occasional smoker	4.21	1.24–14.26	0.021*
	Passive smoker	0.80	0.16–3.93	0.781
Previous medical condition before college	No			
	Yes	2.10	1.10–4.00	0.024*
Previous mental health condition before college	No			
	Yes	2.61	1.35–5.04	0.004*

*Note:* * Means significant *p* value.

## 4 Discussion

The current study through a cross‐sectional design found that clinically significant health anxiety was present in 77/424 health profession students (18.2%; 95%CI, 14.8%–22.1%) in Riyadh, declaring that nearly one in five students scored at or above the SHAI cutoff which is extremely important because health anxiety may interfere with wellbeing, academic functioning, reassurance‐seeking behavior, and healthcare use, and it has been recognized as a meaningful worry in medical and health professions education [2, 3].

The observed prevalence is broadly consistent with prior Saudi and regional evidence showing that illness‐related concerns are common among medical students and health profession learners. A study from western Saudi Arabia reported illness anxiety disorder in 17% of medical students, a proportion close and consistent to the 18.2% observed in the present study [13]. Similar health anxiety and hypochondriacal concerns have also been reported in student samples from Pakistan, India, Peru, and the United Arab Emirates [14, 15, 17, 18]. These similarities suggest that health anxiety may be a recurring issue across different health profession programs, notably reflecting frequent exposure to disease‐related content, clinical environments, and heightened self‐monitoring of bodily sensations.

Female students had significant higher mean SHAI scores than male students across the continuous SHAI outcomes, where for total SHAI score, females scored 18.1 ± 8.8 compared with 14.9 ± 8.8 among males (*p* = 0.006) and this pattern aligns with broader literature showing that women may report higher anxiety symptom burden in some educational contexts [9, 10, 18]. However, sex was not significantly associated with clinically significant health anxiety in the categorical chi‐square analysis (*p* = 0.155) and was not shown as a significant predictor in the adjusted logistic regression model, and the discrepancy between continuous and categorical findings suggests that sex may affect symptom intensity more than the likelihood of crossing the SHAI clinical cutoff.

On discussing the previous medical and mental health conditions among the students, they appeared as the strongest and most consistent predictor of health anxiety where with a previous medical condition had higher mean total SHAI scores (21.7 ± 10.0 vs. 16.7 ± 8.4; *p* < 0.001), a higher prevalence of clinically significant health anxiety (36.5% vs. 14.3%; *p* < 0.001), and higher adjusted odds in the logistic regression model (adjusted OR = 2.10; 95% CI, 1.10–4.00; *p* = 0.024). Similarly, students with a previous mental health condition had higher mean SHAI scores (22.9 ± 10.2 vs. 16.6 ± 8.3; *p* < 0.001), a higher prevalence of clinically significant health anxiety (40.9% vs. 14.0%; *p* < 0.001), and the strongest adjusted association (adjusted OR = 2.61; 95%CI, 1.35–5.04; *p* = 0.004). These findings are plausible because previous illness experiences may increase attention to bodily symptoms, illness threat, and reassurance‐seeking, which are central features of health anxiety [2, 3]. Prior mental health difficulties may also increase vulnerability to anxious interpretation of physical sensations and may reflect a broader tendency toward psychological distress [13, 14, 15, 16, 17, 18].

Choosing a college because of a close relative's medical or mental health condition was also associated with health anxiety. Students in this category had the highest mean total SHAI score (23.2 ± 9.2), and clinically significant health anxiety was present in 50.0% of them compared with 15.1% among those who selected their college because of personal interest (*p* < 0.001). In the adjusted model, choosing college because of a close relative's medical or mental health condition remained significantly associated with clinically significant health anxiety compared with personal interest (adjusted OR = 2.98; 95%CI, 1.03–8.65; *p* = 0.044). This may reflect increased exposure to illness within the family, stronger salience of health‐related threat, or personal identification with caregiving roles and medical careers through family experience. However, the exact pathway cannot be determined in a cross‐sectional design.

Smoking status showed an association with health anxiety, with occasional smokers having the highest risk profile. Occasional smokers had the highest mean total SHAI score (23.5 ± 0.6), and 53.8% had clinically significant health anxiety compared with 16.3% of nonsmokers (*p* = 0.005). In logistic regression, occasional smoking was associated with higher odds of clinically significant health anxiety compared with nonsmoking (adjusted OR = 4.21; 95%CI, 1.24–14.26; *p* = 0.021). This result should be interpreted cautiously because the occasional smoker subgroup was small (*n* = 13), resulting in a wide confidence interval and limited precision. Nevertheless, the finding is consistent with evidence that smoking‐related behaviors can co‐occur with anxiety symptoms and may reflect maladaptive coping or stress‐regulation patterns [17].

Some variables were associated with higher continuous SHAI scores but were not significantly associated with clinically significant health anxiety in categorical analyses. Having a first‐degree relative who is a doctor was associated with higher total SHAI scores (19.2 ± 9.3 vs. 16.9 ± 8.7; *p* = 0.015), but it was not significantly associated with clinically significant health anxiety (*p* = 0.072). Similarly, physician visit during the preceding 6 months was associated with higher total SHAI scores (19.0 ± 8.7 vs. 16.5 ± 8.9; *p* = 0.006), but the categorical association with SHAI ≥ 27 was not statistically significant (*p* = 0.082). This suggests that healthcare exposure or healthcare‐seeking may increase symptom awareness or average health anxiety scores without necessarily pushing students above the clinical cutoff. Interpretation should also avoid over‐pathologizing appropriate healthcare use, as some physician visits may reflect genuine health needs.

College showed small differences in continuous SHAI scores, with the highest total mean among pharmacy students (22.8 ± 10.9; *p* = 0.036), and the negative consequences subscale also differed across colleges (*p* = 0.023). However, college was not significantly associated with clinically significant health anxiety in the categorical analysis (*p* = 0.138), and some college subgroups were small. Academic level and marital status were not significantly associated with SHAI scores or clinically significant health anxiety. Overall, these findings suggest that health anxiety in this sample was linked more strongly to prior illness experiences, health‐related family background, and occasional smoking than to academic year or marital status.

From an educational and student‐support point of view, the findings declare the importance of not relying on academic performance markers when identifying students at risk, and hence screening and counseling initiatives may benefit from paying attention particularly to students with previous medical or mental health conditions, and students who report persistent health‐related worries. Future work should use longitudinal and mixed‐method designs to clarify causal relationships and explore how much health profession students experience and interpret health‐related information in the educational process.

### 4.1 Strengths and Limitations

This study had several strengths, and a major strength of them is that the study included students from several health profession colleges rather than a single discipline, which improves the internal breadth and variability of the sample and allows comparison across different educational backgrounds. Also, the use of the SHAI, a validated and widely used instrument for assessing health anxiety in clinical and nonclinical populations [2, 3], in addition to the analysis, which examined continuous SHAI scores, categorically clinically significant health anxiety, and adjusted logistic regression results, providing a more nuanced picture of associated factors. At the same time there are some limitations like the cross‐sectional design which allows identification of associations but using binary logistic regression analysis and adding effect sizes change the study to analytical one giving more strength to the results and its impact. Despite the convenience sample came from a single university and was predominantly female, it does not limit generalizability to all health profession students in Saudi Arabia as most of the universities have similar male‐to‐female distribution. Self‐reported data may also be affected by recall bias, particularly for sensitive variables such as mental health history and smoking status, but the anonymous questionnaire and the right to withdraw without penalty could overcome this issue.

## 5 Conclusion

Clinically significant health anxiety was identified in nearly one in five health profession students in this single‐university cross‐sectional study. Previous medical or mental condition, occasional smoking, and choosing the college because of a close relative's medical or mental health condition were independently associated with clinically significant health anxiety. The findings highlight the importance of student‐centered mental health awareness, screening, and supportive counseling services for health profession students, especially those with prior personal or family health‐related experiences. Accessible mental health awareness programs should be provided by universities and health profession colleges in addition to counseling services, and referral pathways for students with clinically significant health anxiety or persistent health‐related concerns. Future research studies should use a multicenter approach and apply longitudinal or mixed method designs to understand variation in health anxiety over time and to help formulate preventive measures.

## Author Contributions

**Eman Dawood:** conceptualization, writing – original draft, methodology, validation, writing – review and editing, formal analysis, supervision. **Agnes Monica:** conceptualization, writing – original draft, writing – review and editing. **Alyia Abdullah:** conceptualization, methodology, writing – original draft, writing – review and editing. **Ghada Alalyani:** conceptualization, writing – original draft, methodology. **Nada Alzhrani:** conceptualization, writing – original draft, methodology. **Fai Alqahtani:** conceptualization, writing – original draft, methodology. **Ghadeer Bin Ali:** conceptualization, writing – original draft, methodology. **Hanaa Abo Shereda:** conceptualization, writing – original draft, writing – review and editing.

## Funding

The authors have nothing to report.

## Ethics Statement

Approval from the research unit at the College of Nursing, Riyadh, King Saud bin Abdulaziz University for Health Sciences, was obtained. Ethical approval from the Institutional Review Board Committee at King Abdullah International Medical Research Center (KAIMRC) was granted (SP23R/242/11). All participants provided informed consent and were informed about voluntary participation, confidentiality, anonymity, and their right to withdraw without penalty. All methods were carried out in accordance with relevant guidelines and regulations.

## Conflicts of Interest

The authors declare no conflicts of interest.

## Author Approval and Guarantor Statement

All authors have read and approved the final version of the manuscript. Dr. Eman Dawood had full access to all of the data in this study and takes complete responsibility for the integrity of the data and the accuracy of the data analysis.

## Transparency Statement

This manuscript is an honest, accurate, and transparent account of the study being reported; that no important aspects of the study have been omitted; and that any discrepancies from the study as planned have been explained.

## Data Availability

The data that support the findings of this study are available on request from the corresponding author. The data are not publicly available due to privacy or ethical restrictions. The author confirms that all data collected and analyzed during this study are included in this manuscript. Additional de‐identified participant‐level data may be made available by the corresponding author upon reasonable request and subject to institutional approvals.
